# Parent's Relative Perceived Work Flexibility Compared to Their Partner Is Associated With Emotional Exhaustion

**DOI:** 10.3389/fpsyg.2018.00640

**Published:** 2018-05-03

**Authors:** Constanze Leineweber, Helena Falkenberg, Sophie C. Albrecht

**Affiliations:** ^1^Stress Research Institute, Stockholm University, Stockholm, Sweden; ^2^Department of Psychology, Stockholm University, Stockholm, Sweden

**Keywords:** autonomy, couple level, emotional exhaustion, flexibility, gender, work-time control

## Abstract

A number of studies have found that control over work conditions and hours is positively related to mental health. Still, potential positive and negative effects of work flexibility remain to be fully explored. On the one hand, higher work flexibility might provide better opportunities for recovery. On the other hand, especially mothers may use flexibility to meet household and family demands. Here, we investigated the association between parent's work flexibility, rated relative to their partner, and emotional exhaustion in interaction with gender. Additionally, gender differences in time use were investigated. Cross-sectional analyses based on responses of employed parents to the 2012 wave of the Swedish Longitudinal Occupational Survey of Health (SLOSH) were conducted (*N* = 2,911). Generalized linear models with gamma distribution and a log-link function were used to investigate associations between relative work-flexibility (lower, equal, or higher as compared to partner), gender, and emotional exhaustion. After control for potential confounders, we found that having lower work flexibility than the partner was associated with higher levels of emotional exhaustion as compared to those with higher relative work flexibility. Also, being a mother was associated with higher levels of emotional exhaustion, independent of possible confounders. An interaction effect between low relative work flexibility and gender was found in relation to emotional exhaustion. Regarding time use, clear differences between mothers' and fathers' were found. However, few indications were found that relative work flexibility influenced time use. Mothers spent more time on household chores as compared to fathers, while fathers reported longer working hours. Fathers spent more time on relaxation compared with mothers. To conclude, our results indicate that lower relative work flexibility is detrimental for mental health both for mothers and fathers. However, while gender seems to have a pronounced effect on time use, relative work flexibility seems to have less influence on how time is used. Generally, mothers tend to spend more time on unpaid work while fathers spend longer hours on paid work and report more time for relaxation.

## Introduction

Many parents suffer from insufficient recovery, which in turn may lead to feelings of being overextended and depleted of one's emotional and physical resources (Maslach et al., 2001), i.e., feelings of emotional exhaustion—one of the basic stress dimension of burnout. One possible underlying factor for parental exhaustion might be the experience of work-family conflict. While a number of cross-sectional studies have reported associations between work-family conflict and exhaustion (Allen et al., 2000; Amstad et al., 2011), fewer studies have investigated the relationship between work-family conflict and feelings of exhaustion using a longitudinal design. Evidence from longitudinal studies is mixed and although it has been found that work-family conflict is related to subsequent feelings of exhaustion over time (Innstrand et al., 2008; Leineweber et al., 2013), other studies report reversed (Richter et al., 2015), or no (Rantanen et al., 2008) relationship. To increase parent's possibilities to combine work and family demands, i.e., to reduce their perceived work-family conflict, employers often introduce flexible work arrangements. Indeed, increased work time control could be one appropriate measure to decrease feelings of work-family conflict; a systematic review found moderately strong evidence for a causal relationship from work time control to work-family conflict (Nijp et al., 2012).

The daily life of parents, as for working individuals in general, is largely determined by working hours (Fagan, 2001; Fagan and Burchell, 2002; Costa et al., 2004). Working hours can concern the number of work hours, the timing of work hours, and the degree of work time autonomy (Fagan, 2001). A number of studies have shown that all these components can impact workers' health, including levels of burnout and exhaustion (Grzywacs et al., 2008), directly and via effects on work-life balance (van Amelsvoort et al., 2004; Virtanen et al., 2012; Kivimäki et al., 2015; Albrecht et al., 2017). For instance, long working hours limit how much spare time is left and can increase feelings of work-family conflict (Jansen et al., 2004) and thereby ill-health. Family and social life can be affected by timing of work hours, for instance in the case of early morning, late evening, or night-time shifts (van Amelsvoort et al., 2004). Work-time autonomy, which includes control over the number as well as scheduling of hours worked, has been related to time adequacy (fitting family or social commitments outside work) for women, and to overtime and work intensification for men (Lott, 2015). Work-time autonomy has also been positively related to mental health (Ala-Mursula et al., 2005; Salo et al., 2014).

While many studies on work flexibility regard solely the *time* when to work, today we can also observe an increasing flexibility regarding the *place* where the work is conducted. Different terms are used to describe this flexibility and terms such as telework, telecommuting, distance work, and flexible work place are used more or less interchangeable (Allen et al., 2015). Telework has been shown to have many benefits, such as better possibilities to combine work with family demands, less time spend on commuting, and fewer disruptions in work from colleagues. However, the flip side of the coin are blurred boundaries between work and family with potentially increased difficulties to switch off from job-related thoughts and consequently feelings of stress and impaired sleep and recovery (Beckers et al., 2012; Golden, 2012).

When children are living at home and need care, parents are interdependent of each other to provide such care. The work arrangements of one partner influence not only her- or himself, but also her or his partner (Nock and Kingston, 1984; Grzywacs et al., 2008). Still, most research focuses on the individual employee, and disregards the partner's situation. To the best of our knowledge, research on the role of relative work flexibility in comparison to the partner is scarce. The present paper is the first to investigate the association between parents' relative work flexibility and emotional exhaustion in interaction with gender. Work flexibility is measured in comparison to the partner and thus allows to investigate the relative effect of flexible work on emotional exhaustion. Further, we study how time use differs between women and men in relation to their relative work flexibility.

The Effort-Recovery Theory (Meijman and Mulder, 1998) provides a theoretical framework for our study. According to this theory the key determinants of balance between effort and recovery are workload and work control. From a health perspective, high workload may adversely influence the effort-recovery balance. Workload is determined to a large extent by the amount and complexity of work, but also by temporal aspects of work (i.e., working-time arrangements). The number and distribution of work hours determines the duration and intensity of exposure to workload, as well as opportunities for recovery. Prolonged exposure to high work demands may lead to sustained psycho-physiological activation and a disrupted recovery process. Work flexibility can be assumed to promote a favorable balance between effort and recovery as it facilitates recovery opportunities during work by means of control over breaks and between duties. This enables employees to adjust their work and recuperation times to their current need for recovery. Other key occupational health theories (i.e., the Self-Determination Theory) (Deci and Ryan, 1985), the Job Characteristic Model (Hackman and Oldham, 1976), and the Job Demand-Control Model (Karasek and Theorell, 1990) suggest that work flexibility, as a sub-dimension of general autonomy, may promote workers' motivation, health and performance, and may reduce stress and feelings of exhaustion. Work flexibility influences these outcomes by means of at least two processes: (i) through the actual utilization of work flexibility and associated changes in working times and related work characteristics; and (ii) by the mere opportunity to self-determine working arrangements that may stimulate favorable outcomes, irrespective of an actual change in working times (Beckers et al., 2012; Deci et al., 2017). Finally, based on theories of work-home interaction, (Geurts and Demerouti, 2003) work-flexibility promotes a better work—non-work balance (and thus mitigates stress and exhaustion) because it enables employees to adjust their work hours and arrangements to meet obligations, needs, and activities in their private life.

Although these theoretical assumptions point toward a positive effect of increased work flexibility on health and job related outcomes, it is also possible that it may have negative effects. Highly flexible work arrangements are more common in high-level positions which are associated with longer working hours and less time for recovery. Furthermore, if people choose “unhealthy” work schedules, such as compressed work schedules, in order to satisfy their social needs or maximize their free time, work flexibility could engender insufficient recovery and thus have a negative impact on health. While research on telework has elucidated positive as well as negative consequences, research on work-time flexibility has tended to emphasize the positive sides. Still, evidence that work-time flexibility is positively associated with health and well-being remains inconsistent and limited (Nijp et al., 2012), at least in part due to mostly cross-sectional studies and diverging measures of work-time flexibility. Nonetheless, most studies have found positive associations between work flexibility and health. Such could Bond et al. (2002) show that employees with more access to flexible work arrangements reported fewer mental health problems. A different study found that increased or stable high levels of work-time flexibility were associated with longer sleep duration, less fatigue, and less depressive symptoms after 1 year (Takahashi et al., 2012). Likewise, Kubo et al. (2013) found that the combination of high work-time flexibility and low variability of work schedules (more regular working times) was associated with better sleep quality, fatigue recovery, and work-life balance.

The picture of how work flexibility, relative to once partner, relate to emotional exhaustion is not evident. However, comparisons regarding work flexibility may impact on well-being in several ways. On the one hand, recognizing low work flexibility may have a direct effect on mood. On the other hand, having more work flexibility than the partner might mean higher pressure to handle family demands, such as leaving and fetching children at preschool or school etc. and consequently fewer opportunities to recover/switch off. Still, more flexibility facilitates handling such demands. As most previous studies have found that more work flexibility is positively associated with better health and work-life balance, we form the following Hypothesis:

*Hypothesis 1: Relative work flexibility will be associated with symptoms of emotional exhaustion, so that lower flexibility relatively to the partner will be positively associated with symptoms of emotional exhaustion*.

Generally, women are found to report slightly higher levels of emotional exhaustion than men (Puranova and Muros, 2010). The reason for this is still to be uncovered and different theories have been suggested to explain gender differences in health. These theories can be divided into biological explanations (i.e., evolutionary and psychoanalytical theories) and social theories (i.e., social role theory and social construct theory) (for an overview see Malach-Pines and Ronen, 2016). Both perspectives suggest gender differences in burnout, although they differ regarding expected universality and size of gender differences. Social theories suggest that gender roles of women and men are associated with differences in exposure to demands and obligations. In line with this *different exposure Hypothesis*, gender differences in health are reduced when sector, occupation, and position are considered (Emslie et al., 1999; Falkenberg et al., 2015). Indeed, it has been found that the relation between job demands and health-related outcomes are similar among women and men (Theorell et al., 2015; Sverke et al., 2016), but that women generally report higher levels of demands (Sverke et al., 2016) and job strain (Theorell et al., 2014). Based on these theoretical and empirical considerations, our second Hypothesis reads:

*Hypothesis 2: Being a mother will be positively associated with symptoms of emotional exhaustion compared with being a father*.

It remains unclear who benefits most and under which conditions from work flexibility. It has been suggested that the family is a more important sphere for women while work is a more important area for men (Pleck, 1977; Greenhouse and Beutell, 1985; Wiley, 1991). Based on this traditional gender-role model, fathers would fulfill their family identity by being breadwinners while the mother's family identity involves domestic and family obligations (Wiley, 1991). Consequently, mothers would benefit more from flexible work arrangements than fathers (Hill et al., 2008), as higher flexibility facilitates handling work and family spheres. On the other hand, it may be equally plausible that fathers benefit more from flexible work arrangements than mothers because they may more often utilize this flexibility to create times of leisure. In contrast, mothers may use work flexibility to increase activity at children's schools or their community (Grzywacs et al., 2008). Another perspective is that in a similar situation, mothers and fathers would be affected in a similar way. Based on this egalitarian role balance model (Schieman et al., 2006) mothers and fathers with the same amount of flexibility would report the same levels of emotional exhaustion.

Only few studies have investigated if work flexibility and hence the possibility to adapt work to private life benefits the genders differently. A study with a multi company US sample has found that women generally valued flexible work arrangements more than men—especially during phases of life with children living at home (Hill et al., 2008). One study, using a Swedish sample of 800 employees who were living with a partner and/or children, found that for women, very high levels of control at work could reduce work-family conflict. For men, more control (irrespectively of the level) was beneficial to reduce work and family conflict (Grönlund, 2007). In a sample of 10,608 participants from 21 countries, higher levels of control over work schedules were found to be linked to less work–family conflict, especially for women (Lyness et al., 2012). Fewer studies have looked upon health outcomes, but some findings from the Finnish 10-Town study suggest that women benefit more from work flexibility (in terms of sickness absence and self-rated health) than men (Ala-Mursula et al., 2002, 2004, 2006), although these results may be biased by the gender imbalance in the study sample. Based on the empirical finding our next Hypothesis reads:

*Hypothesis 3: Relative work flexibility interacts with gender in association to emotional exhaustion, so that flexibility reduces emotional exhaustion more for mothers than for fathers*.

Even though the differences in gender roles seem to becoming less dominant and gender differences in time use became more equal during the past years, women still spend more time on home and household work (Noonan et al., 2007; Statistics Sweden, 2014). Consequently, it has been suggested that women use work flexibility to engage in additional family-related activities whereas men may use this kind of arrangement for personal leisure (Grzywacs et al., 2008) or to increase work time (Lott, 2015). How parents use their relative work flexibility affects their possibilities to recover, and in the longer run, their level of exhaustion. Thus, we also wanted to investigate the following research question:

Research question 1: How do mothers and fathers use their relative work flexibility in terms of family demands and relaxation?

## Materials and methods

### Study population

Data was derived from the Swedish Longitudinal Occupational Survey of Health (SLOSH) study. SLOSH is a longitudinal cohort study with a focus on associations between work life participation, work organization, work environment, social situation, health, and well-being. The SLOSH sample consists of all respondents to the Swedish Work Environment Surveys 2003-2011. All labor market sectors and occupations are represented, and the number of women and men is approximately equal. Participants are invited every second year to respond to a postal questionnaire in two versions: One for those currently in paid work and one for those permanently or temporarily outside the labor force. For details see elsewhere (Magnusson Hanson et al., 2018). The current study is based on data drawn from wave 4 (year 2012), as the predictor variable was measured at this occasion and not before or after. In 2012, a total of 9,880 individuals responded to SLOSH (response rate 57%). The current study population was restricted to independent individuals in paid work (i.e., no couples) (*n* = 7,325) with children living at home (*n* = 3,428), who had valid information on the predictor and the outcome variable and indicated living with a partner (*n* = 2,911). In average, participants were 45.9 years old (*SD* = 7.5). 1587 (54.2%) were mothers and 1,333 (45.8%) were fathers. A majority (*n* = 1,490; 51.2%) reported having 2 or 3 children living at home. More than half of all participants (*n* = 1,566; 53.8%) had post-secondary education. What regards the socio-economic position, 740 (26.3%) parents were manual workers, 1,356 (47.7%) were lower or intermediate non-manual employees and 725 (25.5%) were professional or upper-executive non-manual employees. The average yearly income was 371,700 Swedish crowns (*SD* = 188,800). The SLOSH study has been approved by the Regional Research Ethics Board in Stockholm and all participants gave their informed consent.

### Measures

#### Emotional exhaustion

Emotional exhaustion was measured by 5 items of the Emotional Exhaustion subscale of the Maslach Burnout inventory, General Survey (MBI-GS) (Maslach et al., 1996). Answers were given on a response scale with six options (as compared to seven response options in the original scale) reaching from 1 = “Every day” to 6 = “A few times a year or less/Never.” Items were reversed, so that higher values indicated more symptoms of emotional exhaustion, and a mean was calculated if responses to not more than 2 items were missing. Cronbach's alpha was 0.88.

#### Relative work flexibility

Work flexibility relative to the partner was measured by one item: “When you compare your and your partner's ability to decide over your work situation, e.g., working hours and whether you could work from other places than the actual workplace, how do you rate your flexibility?” Responses covered 6 options. Those who answered positively on the first option (1 = “I don't have a partner”) were omitted from analyses and the remaining were categorized as followed: 2 = “My partner is much more flexible” and 3 = “My partner is somewhat more flexible” into “more flexible partner,” 4 = “Me and my partner are rather similarly flexible” into “equal flexible,” and 5 = “I am somewhat more flexible” and 6 = “I am much more flexible” into “less flexible partner.”

#### Gender, time use, and covariates

A binary measure of gender was obtained from register data linked to questionnaire responses by means of the unique Swedish ten-digit personal identification number. As this study was restricted to parents we use the terms “mothers” and “fathers” onwards.

Time spent on different activities was measured by one question derived from Lundberg et al. (1994) asking “Estimate your total workload except gainful employment (i.e., home- and household chores, care of children, and other duties) for 1 week. Imagine a normal work week (7 days).” After that introductory question a number of activities were provided, i.e., commuting time to work, home and household chores, children-related activities, caregiving, other duties (e.g., take care of grandchildren, taking care of pets), leisure-time activities, and time for relaxation. Responses were given on a scale with five response options (0 h/week; 1–5 h/week; 6–10 h/week; 11–15 h/week; >15 h/week). To construct a continuous scale, responses were recoded into the median value of each category, i.e., those who answered having spent 1–5 h/week on a certain activity were given a value of 3 h/week. The highest category (i.e., >15 h/week) was coded as 18 h/week. Weekly working hours were measured by one item with seven response alternatives covering 10 h each, ranging from' <10 h/week' to'>=60 h/week'. For analyses responses were recoded similarly to non-work activities (e.g., 10–19 h/week into 15 h/week). The highest category (60 h or more/week) was recoded as working 65 h/week.

We included age, income, socio-economic position, working hours, shift work, overtime, and work-time control as covariates. Age was measured in years and obtained from register data linked to questionnaire responses. Income was derived from income and taxation register (IoT) data and regards the total earned income in 2010 before taxes (in thousands of Swedish crowns). A measure of socio-economic position was based on the Swedish socio-economic classification and re-coded into 1 = manual workers, 2 = non-manual employees, 3 = professionals and upper-executive non-manual employees. Shift work was measured by a single item with nine response options and dichotomized into not working shifts (i.e., daytime work, afternoon and evening work, non-regulated working hours, and other working hours) or working shifts [i.e., night work, rotating shift work (with and without night work), roster work (with and without night work)]. Overtime was measured by single question “Do you work overtime at least once a week?” answered with “yes” or “no.” Work-time control was measured using an adapted 6-item questionnaire from Ala-Mursula (Ala-Mursula, 2006; Albrecht et al., 2016) covering the perceived influence over: the length of the duty period; start and finish times of the duty period; which days to work; taking breaks at work; running private errands during work time; and scheduling vacation and other leave. Level of control was rated on a 5-point Likert scale ranging from 1 = very little to 5 = very much. Two subscales were derived measuring control over daily hours (i.e., the mean of the items “influence over length of duty period” and “influence over start and finish times”) and control over time off (i.e., the mean of the items “influence over taking breaks,” “influence over scheduling leave,” and “influence over running private errands”). The item “which days to work” was not included in either sub-dimension (for details see Albrecht et al., 2016).

### Statistical analysis

All analyses were run in SAS 9.4. Chi-square tests and analyses of variance (ANOVAs) were performed to assess differences in possible confounders between groups of relative work flexibility and gender. Associations between relative work flexibility and emotional exhaustion were estimated using generalized linear models using the genmod procedure with gamma distribution and log-link function. Multivariable models were calculated in several steps. Following separate unadjusted models for relative work flexibility (Model 1a) and gender (Model 2a), we added possible confounders to each separate model (Models 1b and 2b). In a next step, we calculated interactions between work flexibility and gender (Model 3a). Finally, we controlled the full model for age, income, socio-economic position, working hours (as time categories), shift work, overtime, and work-time control (Model 3b). Analyses of variance (ANOVAs) with Tukey adjustment were used to assess differences in time use between the 2 (gender) ^*^ 3 (relative work-flexibility) groups.

## Results

### Descriptive statistics

Results revealed some differences between mothers and fathers in regard to background factors. Generally, fathers were slightly older (*M* = 46.66 years, *SD* = 7.73 vs. *M* = 45.34 years, *SD* = 7.33), had a higher mean income (*M* = 435.08 SEK, *SD* = 214.45 vs. *M* = 317.99 SEK, *SD* = 143.54), had more often a professional or upper-executive position (30.34 vs. 21.85%), worked more often overtime (48.52 vs. 40.04%), and experienced higher control over daily hours (*M* = 3.18, *SD* = 1.30 vs. *M* = 2.73, *SD* = 1.30) and control over time off (*M* = 3.53, *SD* = 0.92 vs. *M* = 2.99, *SD* = 1.06) as compared to mothers. Mothers reported more often working shifts (14.64 vs. 10.01%) as compared to fathers. Also, 48% of all fathers in the sample reported having a partner with less work flexibility, while the same was true for 24% of mothers.

Means and percentages for the study variables in relation to gender and relative work flexibility are presented in Table 1. Both among mothers and fathers differences in income between groups of relative work flexibility were found. Among fathers, those who reported having *more* work flexibility as compared to their partner had the highest income, and those who reported *equal* flexibility had higher income than those who reported having *less* work-time flexibility as their partner. Among mothers, those who reported having relatively more work flexibility had a statistically significant higher income than those reporting having *less* relative work flexibility as compared to their partner. Also, both among mothers and fathers, being more flexible than the partner was more common among those with a professional or upper-executive position. Regarding working hours, among fathers, those who reported having more work flexibility as their partner reported working longer hours. Among mothers, no association between relative work flexibility and working hours was found. Both among mothers and fathers, a gradient regarding shift work was found with those reporting relatively high work flexibility working less often shifts. Also, among both mothers and fathers, those who reported to have more work flexibility than their partner worked more often overtime as compared to those having equal or less work flexibility than their partner. Similar relationships were found in regard to control over daily hours and control over time off, indicating that those who reported relatively more work flexibility also reported more control over daily hours and time off. *Post-hoc* analyses revealed significant differences between the three flexibility groups in all variables among both mothers and fathers.

**Table 1:** Means and percentages, respectively, for all variables in the study stratified by relative work flexibility and gender.

	**Women**				**Men**				
	**Group 1: Mothers with a more flexible partner**	**Group 2: Mothers with an equal flexible partner**	**Group 3: Mothers with a less flexible partner**	***p***	**Group 4: Fathers with a more flexible partner**	**Group 5: Fathers with an equal flexible partner**	**Group 6: Fathers with a less flexible partner**	***p***	P^*^
	**41.57% (*n* = 656)**	**34.09% (*n* = 538)**	**24.33% (*n* = 384)**		**16.50% (*n* = 220)**	**35.48% (*n* = 473)**	**48.01% (*n* = 640)**		
Age; mean ±*SD*	45.86 ± 7.40	45.37 ± 7.09	44.42 ± 7.44	0.01	46.66 ± 7.74	47.48 ± 7.76	46.05 ± 7.65	0.01	<0.0001
Income; mean ±*SD*	304.06 ± 134.69	321.54 ± 144.93	336.88 ± 153.83	0.0014	392.94 ± 185.44	425.37 ± 232.95	456.73 ± 206.99	0.0003	<0.0001
Socio-economic position				<0.0001				<0.0001	<0.0001
Manual	23.95 (154)	23.76 (125)	14.48 (54)		46.26 (99)	41.50 (188)	19.61 (120)		
Non-manual	60.50 (389)	51.33 (270)	57.10 (213)		33.18 (71)	33.77 (153)	42.48 (260)		
Professional^**^	15.55 (100)	24.90 (131)	28.42 (106)		20.56 (44)	24.72 (112)	37.91S (232)		
Shift work, % (*n*)				0.0024				<0.0001	0.0003
Yes	17.65 (144)	14.53 (77)	9.71 (37)		20.74 (45)	13.92 (65)	3.79 (24)		
No	82.35 (532)	85.47 (453)	90.29 (344)		79.26 (172)	86.08 (402)	96.21 (609)		
Overtime, % (*n*)				<0.0001				0.0009	<0.0001
Yes	34.46 (225)	40.79 (217)	48.56 (185)		43.12 (94)	43.80 (205)	53.86 (342)		
No	65.54 (428)	59.21 (315)	51.44 (196)		56.88 (124)	56.20 (263)	46.14 (293)		
Control over daily hours (mean ±*SD*)	2.31 ± 1.18	2.78 ± 1.33	3.39 ± 1.18	<0.0001	2.56 ± 1.27	2.89 ± 1.37	3.62 ± 1.11	<0.0001	<0.0001
Control over time off (mean ±*SD*)	2.68 ± 0.96	2.97 ± 1.09	3.55 ± 0.94	<0.0001	3.08 ± 0.93	3.36 ± 0.95	3.81 ± 0.80	<0.0001	<0.0001

* Significance level between genders;

** *Professionals and upper-executive employees*.

### Tests of hypotheses

In Hypothesis 1 we predicted that lower flexibility relatively to partner will be positively associated with symptoms of emotional exhaustion. Results from the general linear modeling (GLM) supported this assumption. As can be seen in Table 2, relative low work flexibility was associated with symptoms of emotional exhaustion (Model 1a), even after controlling for covariates (Model 1b). The association between equal work flexibility and emotional exhaustion found in Model 1a disappeared after controlling for possible confounders (Model 1b).

**Table 2:** Association between relative work flexibility, gender, and emotional exhaustion.

	**Model 1a**		**Model 1b**		**Model 2a**		**Model 2b**		**Model 3a**		**Model 3b**	
	**β**	***p***	**β**	***p***	**β**	***p***	**β**	***p***	**β**	***p***	**β**	***p***
Work flexibility												
Less flexible partner	Ref								Ref		Ref	
Equal flexible partner	**0.047**	**0.023**	−0.012	0.574					0.022	0.444	−0.006	0.834
More flexible partner	**0.160**	<**0.0001**	**0.060**	**0.009**					**0.164**	<**0.0001**	**0.096**	**0.008**
Mother					**0.153**	<**0.0001**	**0.086**	<**0.0001**	**0.142**	<**0.0001**	**0.116**	**0.0001**
Interaction terms												
Mother^*^equal									0.006	0.891	−0.038	0.352
Mother ^*^ partner more flexible									−0.074	0.117	−**0.093**	**0.042**
Age			−**0.004**	**0.001**			−**0.004**	**0.001**			−**0.004**	<**0.001**
Income			−0.000	0.002			−**0.000**	**0.048**			−0.000	0.055
Non–manual^*^			**0.047**	**0.035**			0.028	0.210			0.027	0.230
Professional^*^			**0.130**	<**0.0001**			**0.107**	<**0.001**			**0.108**	<**0.001**
Work hours			0.000	0.983			0.008	0.464			0.009	0.428
Overtime			**0.157**	<**0.0001**			**0.154**	<**0.0001**			**0.153**	<**0.0001**
Shift work			−0.019	0.513			−0.020	0.476			−0.025	0.393
Control over daily hours			−**0.136**	<**0.0001**			−**0.131**	<**0.0001**			−**0.130**	<**0.0001**
Control over time off			−0.004	0.720			−0.007	0.471			−0.005	0.617

* *Reference group is “manual worker.” Bold numbers indicate statistically significant results*.

In line with our Hypothesis 2, which predicted that being a mother will be positively associated with symptoms of emotional exhaustion, gender was associated with emotional exhaustion in all models. Mothers reported higher levels of emotional exhaustion (Model 2a), even after controlling for possible confounders (Model 2b).

To explore a possible interaction between relative work flexibility and gender, an interaction term was introduced in Model 3. We hypothesized that relative work flexibility would reduce emotional exhaustion more among mothers than among fathers. Against our expectation in Hypothesis 3, we found no interaction between gender and work flexibility in the crude model. However, after controlling for covariates an interaction effect among parents reporting relatively lower work flexibility (*p* = 0.042), i.e., fathers with relative lower work flexibility showed a steeper increase in emotional exhaustion as compared to mothers. That is, we could not support our Hypothesis, but found quite the opposite of what we expected.

**Figure 1:**
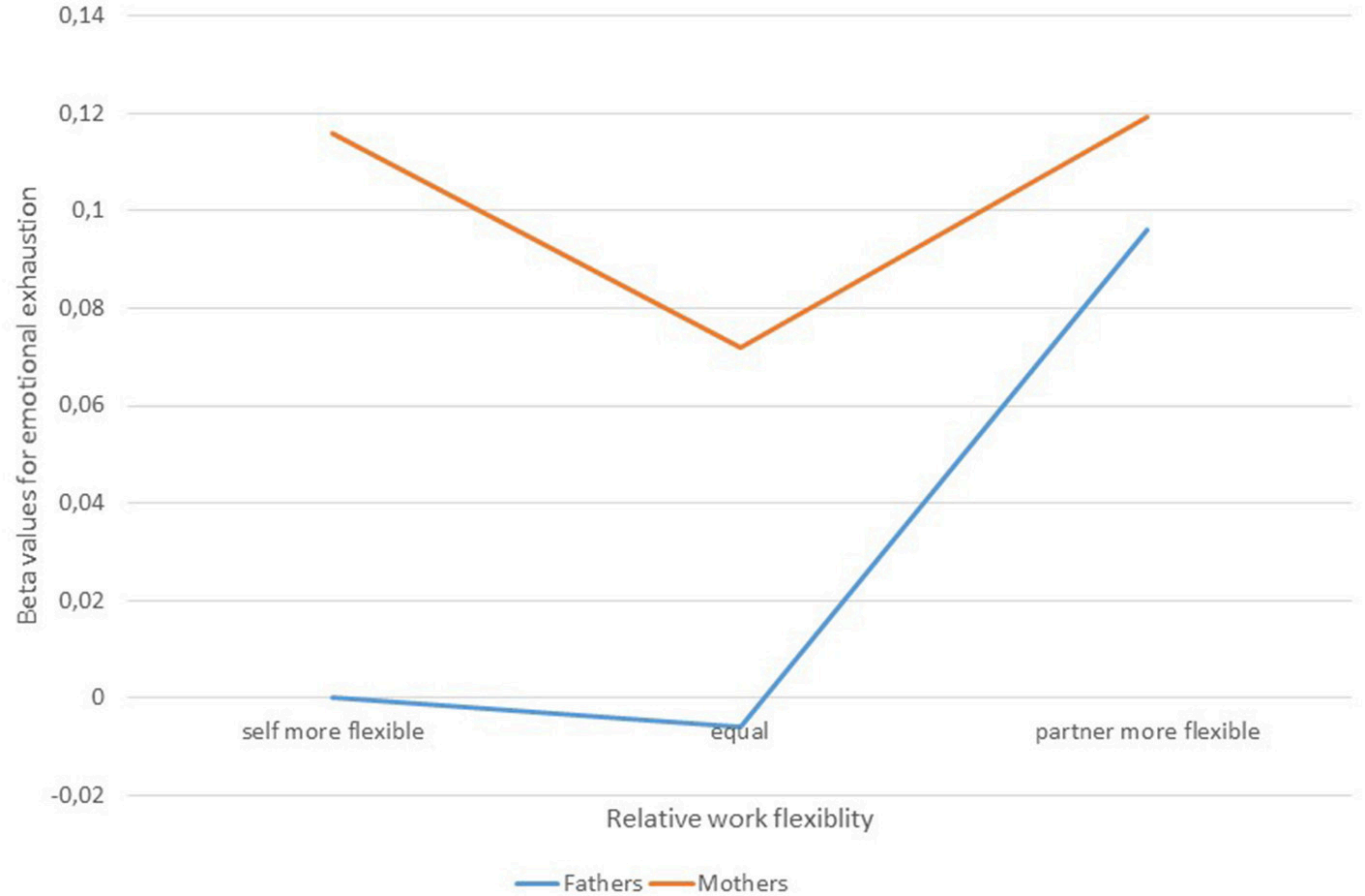
Adjusted risk ratios of emotional exhaustion for mothers and fathers with different relative work flexibility.

Subsequent analyses of combined groups of gender and flexibility, where men with higher work flexibility than their partner formed the reference group, revealed that fathers with less work flexibility than their partner had a slightly increased risk for emotional exhaustion as compared to fathers with relatively high work flexibility (adjusted ß = 0.10, *p* < 0.01). No significant effect of work flexibility was found among mothers (see Figure 1).

### Exploration on differences in time use

In our explorative research question, we wanted to investigate how mothers and fathers use their relative work flexibility in terms of engagement in different activities. Results are presented in Table 3. Analyses revealed that mothers and fathers reported differences in time use. Indeed, statistically significant gender differences were found for all time use measures except time spent on leisure activities. Generally, fathers reported working longer hours in paid work and mothers reported working more hours in unpaid household work, regardless of relative work flexibility. Also, fathers reported to have more time for relaxation. In terms of time spent on home- and household chores, *post-hoc* analyses with Tukey adjustment revealed statistically significant differences between mothers and fathers, but not between work flexibility groups. Also, some statistically significant differences regarding time used for activities with children and care of dependent relatives were found. Generally, mothers reported to spend more time on activities with children and care of dependent relatives. Regarding hours spent on “other duties,” mothers reported longer hours. While no statistically significant differences were found for leisure time activities, several differences between mothers and fathers were found on time for relaxation. Fathers with relatively high work flexibility reported the most hours spent on relaxation—significantly more time than mothers with relatively higher, equal or lower flexibility. Likewise, fathers with equal work flexibility reported significantly more time for relaxation than mothers with equal or relatively lower flexibility. Finally, fathers with relatively lower flexibility had more time for relaxation than mothers with lower flexibility.

**Table 3:** Time use in relation to gender and relative work-flexibility*excluding leisure time and time for relaxation.

	**Women**			**Men**		
	**Group 1: Mothers with a more flexible partner**	**Group 2: Mothers with an equal flexible partner**	**Group 3: Mothers with a less flexible partner**	**Group 4: Fathers with a more flexible partner**	**Group 5: Fathers with an equal flexible partner**	**Group 6: Fathers with a less flexible partner**
Working hours	39.55 ± 8.71^ < 4, 5, 6^	40.55 ± 9.14^ < 4, 5, 6^	40.66 ± 8.68^ < 4, 5, 6^	44.08 ± 7.40^>1, 2, 3^	44.02 ± 8.05^ < 6;>1, 2, 3^	45.46 ± 6.83^>1, 2, 3, 5^
Home- and household work	11.38 ± 4.70^>4, 5, 6^	10.99 ± 4.86^>4, 5, 6^	10.88 ± 4.39^>4, 5, 6^	8.18 ± 4.38^ < 1, 2, 3^	8.14 ± 4.54^ < 1, 2, 3^	8.21 ± 4.48^ < 1, 2, 3^
Children activities	6.31 ± 6.22	6.36 ± 6.11	6.83 ± 6.19^>5^	5.61 ± 5.73	5.40 ± 5.25^ < 1, 2^	5.82 ± 5.61
Care of relatives	0.60 ± .1.88^>5, 6^	0.53 ± 1.97	0.63 ± 2.17	0.33 ± 1.12	0.39 ± 1.21	0.33 ± 1.20^ < 1, 2^
Other duties	2.31 ± 3.70^ < 6^	2.60 ± 4.13^>5, 6^	2.03 ± 3.16	1.97 ± 2.76	1.71 ± 2.88^ < 1, 2^	1.71 ± 3.01^>1; < 2^
Leisure time	4.34 ± 3.13	4.54 ± 3.35	4.64 ± 3.53	4.43 ± 3.54^>1^	4.52 ± 3.19^>1, 2^	4.91 ± 3.56^>1, 2, 3^
Time for relaxation	6.83 ± 4.86^ < 4, 5, 6^	7.06 ± 5.08^ < 5, 6^	7.43 ± 5.18^ < 6^	8.07 ± 5.23	8.01 ± 5.21	8.67 ± 5.33

*The superscript numbers indicate for which between-group comparisons the means differ significantly (p < 0.05). The “ < or >” preceding the superscript numbers indicate the direction of the significant difference in means*.

## Discussion

### Key findings

In this study we investigated if gender and work flexibility, rated relatively, i.e., as compared to the partner, are associated with feelings of emotional exhaustion. Possible interactions between gender and work flexibility regarding emotional exhaustion were also investigated. We found clear associations between emotional exhaustion and low relative work flexibility and gender, respectively. Also, our results indicated an interaction effect between relative work flexibility and gender.

#### Relative work flexibility and emotional exhaustion

In line with our first Hypothesis, both mothers and fathers who reported having relative lower work flexibility as compared to their partner showed more symptoms of emotional exhaustion. This association was irrespective of confounders, including control over daily hours and control over time off, indicating that work flexibility is not only important in terms of absolute flexibility over working hours (as measured by work-time control), but also in relation to the flexibility of the partner.

In a world with changing family structures, most parents still live with a partner. Consequently, the ability to combine work with family demands depends not only on one's own work flexibility and restrictions, but also on the partner's flexibility and willingness to utilize this flexibility to meet work *and* family demands. This interdependence is likely even increasing since gender roles are becoming less clear and an increasing number of mothers and fathers want to have a giving career while at the same time being actively involved in the upbringing of their children. Prior research has indeed shown that couples negotiate work and life strategies as a unit, both to optimize income and take advantage of one partner's work flexibility (Moen and Yu, 2000).

To the best of our knowledge, our study is the first to investigate relative work flexibility. Within work-family research, a few studies have investigated couple-level effects, although these are still widely neglected in the area. For instance, Matias and Fontaine (2015) found that management and planning skills had distinct effects on outcomes between men and women: Men using these skills reduced their own conflict but increased their wives', while women decreased their own conflict and increased their own and their partner's enrichment. In the same line of research our finding provides support for the idea that work flexibility should be investigated on couple-level.

Before including covariates, having equal work flexibility as the partner was associated with more symptoms of emotional exhaustion (see Table 2). This association was no longer significant after including covariates. This indicates that the association between having equal work flexibility and emotional exhaustion found in Model 1 was probably driven by gender differences in covariates, e.g., control over daily hours and control over time off. Indeed, earlier findings show that women often report lower levels of control over working hours—especially the type of flexibility that doesn't come with salary decreases (Ala-Mursula et al., 2005; Albrecht et al., 2016). Similarly, in our study a much larger proportion of mothers (42%) than fathers (16%) reported having a partner with more work flexibility. This is especially interesting considering the fact that women more often make use of flexible work arrangements to combine work and family demands (Hill et al., 2008). That is, although women generally have lower work flexibility, they more often utilize flexibility to be at disposal. In many cases this might be possible only by decreasing working hours (i.e., part-time work), with a related decrease in salary.

#### Gender and emotional exhaustion

We also found support for our second Hypothesis. As expected, women reported generally higher levels of emotional exhaustion than men. Although findings regarding gender and burnout are inconsistent (Malach-Pines and Ronen, 2016), researchers largely agree that women mainly report higher levels of emotional exhaustion. A meta-analysis supports these results finding that women were slightly more emotionally exhausted than men (Puranova and Muros, 2010). It has been argued that gender differences in emotional exhaustion may depend on culture, profession, job, and measures for encountering emotional exhaustion (Malach-Pines and Ronen, 2016).

The assumption that gender differences are explained by differences in the work environment is slightly contradicted by the fact that we found an increased risk of emotional exhaustion among women even after controlling for a number of possible risk factors, including income, socio-economic position, and shift work. This might have to do with possible gender differences in strain at work; previous studies have found higher levels of job strain among women than men in the Swedish population (Theorell et al., 2014). Women work to a large extent in human service and care professions and generally report higher levels of demands (Sverke et al., 2016) and lower levels of work flexibility. Also, we did not take demands from family life into account, which may contribute to differences in emotional exhaustion. Taken together, finding ways to increase mothers work flexibility without salary reduction, as given when e.g., reducing working hours to meet family demands, could be one way to decrease the gender gap in emotional exhaustion.

#### Interaction between relative work flexibility and gender in relation to emotional exhaustion

Theory suggests that work and family-related experiences are gendered and one main interest in our study was to explore a possible interaction effect of gender and relative work flexibility on symptoms of emotional exhaustion. Contradictory to what we hypothesized, we found that relatively low work flexibility seems to be detrimental not to mother's but to fathers' mental health. One possible explanation might be that the relatively low work flexibility is an indicator for a low status rather for men than for women, which may be the reason for the increase in symptoms of emotional exhaustion. No association between relative flexibility and symptoms of emotional exhaustion was found among women. Few other studies have investigated such gendered associations. One study (Grzywacs et al., 2008) that investigated the association between different formal flexitime arrangements (flexitime and/or compressed workweek) and stress and burnout among women and men found that women gained more than men in terms of reduced stress and burnout by flexitime only or compressed workweek only. However, the beneficial effect of having both flexitime and compressed workweeks observed for men was significantly attenuated for women to the extent that women in this arrangement were found to perceive greater stress and burnout than women who were not engaged in any type of formal flexible arrangement.

#### Time use

Regarding time use we found that relative high work flexibility was related to longer working hours, but also more leisure time and time for relaxation among both mothers and fathers. Also, mothers and fathers who reported more work flexibility than their partner spent more time on activities with children than those with relatively low work flexibility. Among women, having relatively high flexibility was also related to less hours spent on home and household chores. Interestingly, among fathers the relationship between relative work flexibility and time spent on home and household chores was the opposite of the association found among women. That is, fathers with relatively higher work flexibility spent more time on household chores than men with relatively lower work flexibility, although differences were non-significant. Still, taken together it seems that mothers experience a limitation in housework reductions that does not apply to fathers, i.e., independent of relative work flexibility mothers spend significantly more time on home and household chores than fathers. These findings are in line with data from Statistics Sweden (SCB) showing a gender gap in unpaid work among parents, which is especially pronounced among parents with young children (Statistics Sweden, 2016). Some differences were found regarding time spent on care of relatives and other demands. Mothers with relatively lower work flexibility spent statistically significant more time on care of relatives than fathers with relatively high flexibility. This is in line with findings suggesting that more daughters than sons provide care for their elderly parents (e.g., in Sweden 57% daughters vs. 43% sons) (Socialstyrelsen, 2013) and that women and those with lower income are more likely to be involved in informal care (Lilly et al., 2007).

We found no significant differences in terms of time spent on leisure activities. However, regardless their relative work flexibility mothers reported having less time for relaxation than fathers.

### Strengths and limitations of the study

Our study is based on a large sample of Swedish parents representing different occupations and social strata. While the results should be generalizable to societies with similar premises, findings might look different in societies with other gender roles and possibilities to combine parenting with employment. The Swedish society has relatively high gender equality—characterized on one hand by a “dual-worker model” in which a majority of both mothers and fathers participate in paid work, but on the other hand by a “dual-carer model” (at least to some extent) in which both parents actively take part in the upbringing of their children (Edlund and Öun, 2016). Thus, our findings might not be generalizable to societies with more traditional gender roles. Especially in societies where mothers and fathers are equally involved in gainful employment while at the same time the mother is expected to do the majority of home and household chores, relative work flexibility may indeed be used differently by mothers and fathers. Future research should look at effects of relative work flexibility in different societies.

To the best of our knowledge, our study is the first to investigate the association between *relative* work flexibility, where parents were asked about their work flexibility in comparison to their partner. This may be considered as a strength, as parents' possibilities and limitations in work flexibility are interdependent of each other. However, data should ideally be collected from both parents and with more than one item to study this interdependence more in-depth. Also, our measure of flexibility does not differentiate between flexibility in working hours and flexibility in place, thus no distinction between these quite different aspects of flexibility can be made. Further, relative work flexibility does not mirror actual work flexibility as it is measured as compared to the partner. It is possible that partners in couples often have relatively equal work flexibility, as flexibility is related to social position (Albrecht et al., 2016) and couples often come from similar social background. Still, our measure of relative work flexibility was associated with both control over daily hours and time off in the expected directions, that is, parents with relatively low work flexibility also reported lower levels of work-time control. Also, our measure of time spent of different activities could not be seen as an exact measure of actual hours spend on each activity, but as an indication of how time was located.

Another limitation is the cross-sectional nature of our study and we cannot exclude the possibility that parents with higher levels of emotional exhaustion experienced lower levels of relative work flexibility, i.e., we cannot exclude a reversed relationship. Still, one recent study rather supported a causal relationship from work-time control to depressive symptoms, while no support for a reversed relationship was found (Albrecht et al., 2017). Future studies employing repeated measurements are warranted to investigate the direction of the relationship.

## Conclusions

Based on a large sample of working parents in Sweden, this study found both relative work flexibility and gender being related to emotional exhaustion. Interestingly, our results further indicate that rather fathers than mothers would gain by having more work flexibility as compared to their partner. Regardless, our findings highlight the importance to consider work flexibility not only on an individual basis, but also relatively on couple-level when evaluating the ability to balance demands from work and home life. Our study also adds support to earlier reports of mothers suffering from emotional exhaustion to a higher extent than fathers. It remains unsolved why this is the case, but our results suggest that gender differences in responsibility for family and household chores as well as in relaxation may be some of the underlying factors.

## Author contributions

CL, HF, and SA formulated the study design. CL carried out the statistical analyses. CL and HF wrote the first and successive drafts of the paper. All authors interpreted the results, revised the text critically for important intellectual content, and approved the final draft of the report. All authors approved of the final version and agree to be accountable for all aspects of the work.

### Conflict of interest statement

The authors declare that the research was conducted in the absence of any commercial or financial relationships that could be construed as a potential conflict of interest.
